# Psychedelic Harm Reduction and Integration: A Transtheoretical Model for Clinical Practice

**DOI:** 10.3389/fpsyg.2021.645246

**Published:** 2021-03-15

**Authors:** Ingmar Gorman, Elizabeth M. Nielson, Aja Molinar, Ksenia Cassidy, Jonathan Sabbagh

**Affiliations:** ^1^MAPS Public Benefit Corp, Santa Cruz, CA, United States; ^2^Fluence, Woodstock, NY, United States; ^3^Depression Evaluation Services, New York State Psychiatric Institute, New York, NY, United States; ^4^Journey Clinical, Inc. Dover, DE, United States; ^5^Todman Psychopathology Lab, Psychology Department, New School for Social Research, New York, NY, United States; ^6^The Center for Attachment Research, Psychology Department, New School for Social Research, Attachment Lab, New York, NY, United States

**Keywords:** psychedelics, harm reduction, psychotherapy, integration, preparation, psilocybin, MDMA, PHRI

## Abstract

Psychedelic Harm Reduction and Integration (PHRI) is a transtheoretical and transdiagnostic clinical approach to working with patients who are using or considering using psychedelics in any context. The ongoing discussion of psychedelics in academic research and mainstream media, coupled with recent law enforcement deprioritization of psychedelics and compassionate use approvals for psychedelic-assisted therapy, make this model exceedingly timely. Given the prevalence of psychedelic use, the therapeutic potential of psychedelics, and the unique cultural and historical context in which psychedelics are placed, it is important that mental health providers have an understanding of the unique motivations, experiences, and needs of people who use them. PHRI incorporates elements of harm reduction psychotherapy and psychedelic-assisted psychotherapy, and can be applied in both brief and ongoing psychotherapy interactions. PHRI represents a shift away from assessment limited to untoward outcomes of psychedelic use and abstinence-based addiction treatment paradigms and toward a stance of compassionate, destigmatizing acceptance of patients' choices. Considerations for assessment, preparation, and working with difficult experiences are presented.

## Introduction

*Psychedelic* means mind-manifesting, and is a broad term applied to compounds and experiences with related subjective effects of an altered perception of one's sense of self and an increased awareness of mental processes. Psychedelic Harm Reduction and Integration (PHRI) is a transdiagnostic and transtheoretical clinical model that incorporates principles of harm reduction psychotherapy, psychedelic-assisted therapy, mindfulness-based modalities, and psychodynamic therapy, and provides a framework for examining and working with psychedelic experiences in clinical care without providing the actual psychedelic experience as part of treatment. PHRI is designed primarily as a psychotherapeutic intervention and is therefore intended to be used by, but not limited to, practitioners for whom psychotherapy is within their scope of practice. Because PHRI, by definition, does not include the prescription or administration of a psychedelic compound, it is not limited to medical professionals. PHRI is intended to guide clinical work with people who have used psychedelics in a variety of contexts, including clinical therapies, spiritual practices, with peer groups, or on their own. Here, we use the word *therapist* to identify licensed providers of psychotherapy who may engage in PHRI as part of their psychotherapy practice. While there are already a variety of clinical psychotherapists offering assistance with the integration of psychedelic experiences based in clinical wisdom and their own theoretical orientations, this is the first attempt we are aware of to provide a cohesive theoretical framework for psychedelic integration in the peer-reviewed literature.

To place PHRI into context, it is useful to understand it in relationship to other approaches used in working with people who use drugs, as well as the background of harm reduction upon which it draws. We can then delineate it from existing approaches and further define its unique therapeutic tasks.

### Approaches to Working With People Who Use Drugs

In 2010, it was estimated that over 30 million people in the United States (US) had at least one psychedelic experience in their lifetime (Krebs and Johansen, 2013). Seventeen percent of those 21–64 years of age have used a psychedelic, and the rate of lifetime psychedelic use was greatest (20%) among people aged 30–34 (total 20%; Krebs and Johansen, 2013). Past-year LSD use grew by 56.4% between 2015 and 2018 (Yockey et al., 2020). Frequent settings for non-clinical psychedelic use include ayahuasca or mushroom ceremonies (Frecska et al., 2016; Lawn et al., 2017; Dorsen et al., 2019), transformational and music festivals (Palamar et al., 2015), and providers of underground psychedelic-assisted therapy (Jade, 2018; Inserra, 2019). This wide variety of available avenues is representative of the population's increased interest.

#### Moral Model of Addiction

Despite these changes in psychedelic use, clinical therapeutic approaches to working with people who use psychedelics have remained stagnant. The moral model of addiction (Pickard, 2017) remains among the dominant ways of understanding drug use. It views people who use drugs as lacking self-control and guilt, and perceives them as having full control over their behavior yet choosing to yield to a weakness (Henderson and Dressler, 2017). This perception has contributed to a highly negative view of people who use drugs and has provided a basis for wide discrimination in the spheres of employment, family relationships, and treatment to name just a few (Henderson and Dressler, 2017; Goodyear et al., 2018).

#### Disease Model of Addiction

In contrast, the disease model of addiction implies that substance use becomes involuntary due to a relapsing neurobiological condition caused by substance-afflicted changes in the user's brain (Polak, 2000; Henderson and Dressler, 2017; Pickard, 2017). Some addiction treatment programs can contribute to shame and stigma through adoption of non-disease models and an abstinence-only approach (Larimer et al., 1999; Tatarsky, 2007), and the resulting reluctance to employ empirically supported treatments (Knudsen et al., 2005; Herbeck et al., 2008; Rieckmann et al., 2011).

In clinical settings, these pathologizing concepts can be misapplied to psychedelic use and become barriers to the inclusion of people who have a relationship with psychedelics, be it harmful or beneficial. As a result, people seeking support for a psychedelic experience could be stigmatized or turned away. This status quo emphasizes the therapists' need for an integrative model for working with psychedelics, substances which do not fit the standard model of drug use because they do not create dependence or addiction as do substances of abuse such as opioids, amphetamines, and benzodiazepines (Nichols, 2016). Additionally, while alcohol, heroin, and cocaine have the highest overall harm scores, those of psychedelics are relatively low (Nutt et al., 2010).

#### Harm Reduction

The harm reduction perspective is an alternative to both the moral and disease models (Szott, 2015). It is based on compassion toward the patient and focuses on reducing the harm caused by substance use, sexual activity, and video gaming (Marlatt, 1996; Marlatt et al., 2011). Harm reduction emphasizes valuing a patient's autonomy and personal rights, respecting their values and preferences, and working to help them expand their options and engage in thoughtful individual decision-making and goal-setting (O'Malley, 1999). Abstinence is not a prerequisite to receive help, which makes this approach uniquely humane (Marlatt, 1996; Marlatt et al., 2011) and efficient in addressing substance misuse (Ritter and Cameron, 2006). However, abstinence is not incompatible with harm reduction, and can be included among the harm reduction goals (Tatarsky, 2003). This paradigm lends itself to the integrated approach in substance use treatment, in which both the substance misuse and potential underlying psychopathology are treated in tandem (Mangrum et al., 2006). This approach may be appropriate for psychedelic users, who may be using to address psychiatric symptoms or promote growth, and who do not usually experience the same negative consequences of substance use on which traditional drug treatment programs focus. These compounds may in fact have anti-addictive properties (Johnson et al., 2014; Bogenschutz et al., 2015; Pisano et al., 2017).

#### Harm Reduction Psychotherapy

Harm Reduction Psychotherapy is a cutting-edge framework that applies harm reduction principles to psychotherapy (Tatarsky, 2007; Tatarsky and Kellogg, 2010; Tatarsky and Marlatt, 2010). One of the most prominent approaches is that of Integrative Harm Reduction Psychotherapy (IHRP; Tatarsky, 2007), one of the key influences for PHRI.

Among the IHRP principles on which PHRI relies are a non-judgmental stance toward substance use and a non-stigmatizing and non-pathologizing approach to the use of drugs, including psychedelics. This represents a shift from traditional abstinence-based paradigms. PHRI also adopts IHRP's approach to the therapeutic alliance (Connors et al., 1997; Coppock et al., 2010) as a key factor in successful work toward reducing drug-use-related risk (Tatarsky, 2007). In addition, IHRP and PHRI cultivate therapist qualities such as flexibility, honesty, warmth, and respect toward the patient, which are associated with positive results in psychotherapy (Ackerman and Hilsenroth, 2003). In IHRP, the therapist-patient relationship is non-hierarchical and non-authoritative. Patient empowerment and compassion toward the patient are crucial aspects of the relationship. The PHRI approach prioritizes the agency and autonomy of the patient; the therapist is there to support the patient in their process.

IHRP incorporates core techniques from relapse prevention, including skills building, active strategizing, urge surfing (Bowen et al., 2014) and exploring many possible meanings of substance misuse for the patient (Tatarsky, 2007). IHRP approaches drug use in the full sociocultural context of a person's life, combining tools from humanistic, cognitive-behavioral, and psychodynamic perspectives to create a work plan uniquely fitted to the needs and circumstances of each patient's situation and that situation's biological, psychological and social dimensions (Tatarsky and Marlatt, 2010). IHRP principles comprise one crucial foundational piece to PHRI; they provide a framework for working outside of an abstinence-only paradigm and for exploring patients' actual experience of the drugs they choose to use, including the possibility that those experiences may be helpful, growth enhancing, and healing.

### Psychedelic-Assisted Psychotherapy

Psychedelic-assisted psychotherapy consists of the administration of a psychedelic in the context of a psychotherapeutic environment and relationship, with the therapist providing psychological support and in some cases specific intervention designed to align with the psychedelic experience and promote change in the target diagnosis. The Multidisciplinary Association for Psychedelic Studies (MAPS; Sessa et al., 2019) is at the forefront of psychedelic research, and is currently conducting Phase 3 clinical trials of 3,4-Methylenedioxymethamphetamine (MDMA)-assisted psychotherapy (“A Phase 3 Program of MDMA-Assisted Psychotherapy for the Treatment of Severe Posttraumatic Stress Disorder (PTSD),”; Noakes, 2019). Psilocybin is currently in clinical trials as a treatment for alcohol use disorder (NCT04410913), major depressive disorder (NCT03181529), and treatment-resistant depression (NCT03775200). Finally, Ketamine is a compound that is legally available with a prescription that has been used in psychedelic-assisted psychotherapy, and providers tend to value both the psychedelic experience and ketamine's antidepressant properties (Dore et al., 2019). While commonalities exist between the types of psychological support and session procedures, the theoretical orientation with which therapists approach their work with patients and its relationship to the treatment diagnosis has varied widely (Sloshower, 2018). Although PHRI implements concepts from psychedelic-assisted psychotherapy, it is distinct in that it explicitly excludes the administration of a psychedelic compound.

### Principles for Working With Non-ordinary States of Consciousness (NOSCs)

Psychedelic experiences have been referred to as NOSCs. In addition to drawing on harm reduction psychotherapy as an approach to working with people who use drugs, PHRI draws on mindfulness-based interventions, psychodynamic therapy, and psychedelic-assisted therapy for understanding and integrating NOSCs in clinical practice.

#### Attitudinal Orientation Toward NOSCs

Mindfulness-based treatment modalities teach meditation practices and skills to apply the resulting insights to reducing stress and distress, including distress associated with mental health and drug use problems (Segal et al., 2012; Bowen et al., 2014; Woods et al., 2019). Mindfulness-based modalities teach an attitude of non-judgmental, open curiosity to whatever the patient experiences, both during formal meditation practice and while going about their life (Kabat-Zinn, 2006; Treleaven, 2018). This attentional and attitudinal shift is thought to result in profound change in how people relate to causes of their distress, thereby resulting in alleviation. PHRI draws on this orientation, considering psychedelic experiences to be another NOSC experience, akin to those achieved through meditation, and asking patients to develop the same sense of non-judgmental curiosity to their psychedelic experiences and all that surrounds them. As in mindfulness-based modalities, it is paramount that therapists practicing PHRI cultivate and embody the same attitudes, so these are explicitly stated and modeled for the patient (McCown et al., 2010; Woods et al., 2019).

#### A Framework for Understanding the Benefit of Loss of Sense of Self

Traditional psychotherapeutic approaches have considered healthy, strong ego functioning to be the central goal of successful therapy, and pathology as the result of an arrest of ego development or a breakdown of its function (Engler, 1984). Our everyday sense of self often shifts and changes in response to environments, relationships, and challenges, and requires a certain degree of flexibility, yet these models do not offer a framework for the healthy development of ego beyond becoming stable and able to cope with life's demands (Engler, 1984). Mindfulness-based modalities, being based in Buddhist psychology and the corresponding understanding of NOSCs, in which the sense of self may be altered or dissolve completely for a time, offer a framework for valuing and working with ego-dissolution and related experiences that may occur under the influence of psychedelics (Nour et al., 2016). In its basic principle, Buddhist psychology conceptualizes the ego as a source of distress and teaches that moving beyond a fixed sense of self reduces that distress, seeing this as a further stage of development (Engler, 1984; Epstein, 1988). While healthy ego functioning is necessary for survival and for moving one toward contemplative practice, it is not seen as the end-goal but rather a stage of development with inherent struggles, beyond which one can ideally move (Engler, 1984). For therapists working with people who use psychedelics, this alternative understanding assists in taking a non-pathologizing approach to working with experiences of ego-dissolution and finding ways that these can be helpful to the patient.

#### Inquiry

*Inquiry*, a component of mindfulness-based interventions, is an improvisational dialogue that consistently facilitates the focus of attention on the participant's experience in a particular way (Crane et al., 2015). Inquiry involves the application of non-judgmental curiosity to communication, enabling the conversation to transpire such that the patient's experience is valued, their intuition honored, and their own autonomy supported (Woods et al., 2016, 2019). Among the key features are focusing on understanding rather than agreement, taking turns in listening with an open mind to avoid a power struggle, actively practicing non-harming speech, and using mirroring techniques. A therapist's engagement in uninvited problem-solving, unsolicited advice, or interpretation of the patient's experience can inhibit the patient's intuitive process and detract from the development of their autonomy (Woods et al., 2016, 2019). The therapist instead focuses on what the patient has tried, what they think and how they feel about the situation, what they see as potential solutions, and what their intuition tells them. The conversation consistently redirects the patient to their own experience such that insights are fostered, developed, and integrated (Woods et al., 2016, 2019). In PHRI, inquiry informs exploration of the psychedelic experience such that insights develop in ways that are consistent with the noetic quality of such experiences (Yaden et al., 2017).

#### Non-directive Approach

Non-directive psychotherapies employ patience and guidance rather than instruction, as instruction relies on a preexisting idea of the underpinnings and best course of solving a specific problem (Snyder, 1945). The non-directive approach has been a core principle of psychedelic-assisted therapies as it enables a patient to adopt an uncritical approach concerning their own psychedelic experience(s) and engage in self-directed insight-finding and meaning-making processes. As such, instead of articulating instructions to a patient, it is appropriate to ask permission before making suggestions to consider or perform an action. Similarly, patients are to determine which aspects of their experience they wish to disclose, hence reinforcing autonomy and personal choice. Therapeutic presence reflects full engagement in the moment-to-moment encounters with patients and establishes a mutual relational connection that builds trust (Geller, 2013).

#### Inner-Directed Approach

The term inner-directed approach refers to the therapist referencing and encouraging the patient to look into their inner experience for insight and solutions.

“Our system is inner-directed… Once we give someone MDMA, they are the ones who produce the content. We follow and support where they are going. Our hypothesis is there is an inner healing intelligence. We all know that's true for our bodies. If you get a scratch or break bones, your body has a mechanism to heal itself. … There is this wisdom of the body to try to sustain itself. We think similarly there is something like that for the psyche.” (Doblin, quoted in Valentino, 2020).

The term *inner healing intelligence* captures this inner-directed approach, which can also be conveyed as intuition or an inner healing capacity (Mithoefer et al., 2008; Clare, 2018; Phelps, 2019). The inner-directed approach is consistent with harm reduction psychotherapy as it values of the patients' autonomy and responsibility for choosing their own goals and course of action.

### Psychodynamic Psychotherapy

Early psychedelic-assisted therapy was strongly influenced by psychoanalytic theory (Grinspoon and Bakalar, 1981). Although psychoanalytic interventions are not a prerequisite for practicing PHRI, certain psychoanalytic theories can be helpful in conceptualizing therapeutic work with individuals who use psychedelics. As mentioned above, having a framework for understanding the “self” is particularly important when working with psychedelic experiences, and psychoanalytic theory offers many such perspectives (Engler, 1984).

Psychedelics may function by dramatically reducing or shifting an individual's defense mechanisms (Fischman, 2019). This can be useful for understanding both a patient's distress during a psychedelic experience and their subsequent alleviation of symptoms. Transference and countertransference may be particularly intensified in PHRI and psychedelic-assisted psychotherapies (Vollenweider and Kometer, 2010; Taylor, 2014; Phelps, 2017). Unlike psychoanalysis, the objective of PHRI is not necessarily to make transference interpretations, but attending to these variables can guide a therapist in their practice. For example, patients may idealize the PHRI therapist, or perceive the therapist as either a withholding gatekeeper of the psychedelic experience or as controlling their capacity to get better. If not carefully attended to, these kinds of appeals to authority can contribute to iatrogenic harm. Additionally, understanding a patient's transference to the psychedelic, whether perceived as a kind of teacher or imbued with the qualities of a caring grandmother, can offer a window into the patient's therapeutic process. Relational psychoanalytic theory can be particular useful in understanding the complexity of transference reactions (Mitchell, 2009).

### Defining and Differentiating PHRI

PHRI is a novel paradigm designed specifically for those who use psychedelics and need clinical support in preparing for or integrating psychedelic experiences. It is rooted in the principles of IHRP and informed by the evidence base of the clinical trials in psychedelic-assisted psychotherapy, yet maintains a focus on the unique needs of people who use psychedelics outside of legal psychedelic-assisted therapy contexts.

PHRI involves supporting exploration and enhancing understanding in patients who develop a relationship with psychedelics without encouragement to use psychedelics, the administration of psychedelics, or the providing of therapy during the psychedelic experience. Therapists can address unique patient needs, such as co-occurring mental health issues, symptoms persisting after psychedelic experiences, and processing potentially overwhelming or disappointing experiences. Therapists can also support patients who seek to sustain the benefits of helpful psychedelic experiences. The goal of integration is to merge the psychedelic experience with the patient's daily life in a way that helps the patient live a fuller life with less distress.

PHRI is not a treatment modality or technique, but serves as a perspective which therapists of all training backgrounds can incorporate into their practice. PHRI is influenced by psychedelic-assisted psychotherapy practices (Mithoefer, 2013; Bogenschutz and Forcehimes, 2017), IHRP (Tatarsky, 2007), relational-psychodynamic theory, and mindfulness-based interventions (Segal et al., 2012; Bowen et al., 2014; Woods et al., 2019). PHRI is consistent with features commonly found across diverse psychotherapeutic orientations such as the establishment of safety, rapport, an empathic presence, agreement on the task of therapy, positive regard, and the authentic presence of the therapist (Feller and Cottone, 2003). Therapists may blend their own theoretical orientations and treatment modalities into the psychedelic integration process to address a specific diagnosis or symptom presentation and consider PHRI an adjunctive approach, as most theoretical orientations and modalities do not have a readily accessible method and rationale for working with psychedelic experiences.

#### Who Might Benefit From PHRI?

Some examples of individuals who might benefit from PHRI are those who might have used a psychedelic on their own in an attempt to resolve a psychiatric symptom; someone who has spontaneously ingested a psychedelic and is consequently experiencing psychological distress; or someone who intends to use a psychedelic but is unaware of the possible risks. The following sections detail therapeutic tasks of PHRI with clinical applications and may guide the therapists' approach to each patient's unique needs. Similarly to psychedelic-assisted therapy, there are some therapeutic tasks fitting to conversations prior to the patient's use of a psychedelic, and some more fitting for afterward. We find it helpful in PHRI to structure sessions as *preparation* sessions and *integration* sessions accordingly.

## Preparation

Like psychedelic-assisted therapy, PHRI can be conceptualized in three stages: preparation, psychedelic experience, and integration. However, a PHRI therapist does not administer nor are they present with a patient during the psychedelic experience. In practice, these components inform and are responsive to each other and to the patient's evolving needs throughout the process. Furthermore, patients sometimes start PHRI after their psychedelic experience, making it necessary for the therapist to adjust their assessment and selection of therapeutic tasks accordingly.

### Assessment

Although it is necessary to utilize Diagnostic and Statistical Manual of Mental Disorders—Fifth Edition (DSM-5; American Psychiatric Association, 2013) or a similar manual depending on the standards of the therapist's region for differential diagnosis in assessment, it can be useful to pose questions specific to PHRI, such as whether a patient's psychedelic experiences were spontaneous or planned. Risks associated with psychedelic use can be minimized when the circumstances provide the potential for a safe, desirable experience (Dalgarno and Shewan, 2005). Still, spontaneous or unplanned psychedelic use in unsupportive circumstances may be of clinical relevance. Therapists may also ask patients about the outcome of their psychedelic experience(s) thus far, and specifically whether the patient has drawn any meaning from them. Psychedelics have been described as having meaning-enhancing properties or evoking a meaning-response which moderates therapeutic outcomes (Hartogsohn, 2018). Participants have reported their first experience with psilocybin in a controlled, clinical setting as being among the most personally meaningful events in their lifetime and of great spiritual significance (Griffiths et al., 2006).

During assessment, the therapist should not presume that psychedelic use indicates a drug use problem or that the presenting symptoms are only indicative of pharmacological side effects. Therapists should instead seek to understand a patient's relationship with psychedelics, especially their motives for use and any resulting overwhelming experiences. This includes taking a psychiatric history, eliciting the patient's reason for seeking assistance, and asking about the patient's history of psychedelic use. Motivations for seeking a psychedelic experience may include recreation, therapeutic healing, spirituality, or personal growth (Winkelman, 2005; Prayag et al., 2015; Dorsen et al., 2019). Inquiring about these additional characteristics among other general inquiries helps to improve the psychedelic integration process and produce meaningful clinical outcomes.

#### Community Involvement

It is also helpful to gauge the patient's connection to a community of psychedelic users (Cavnar, 2014; Dorsen et al., 2019). Such a membership or participation can be nuanced, creating a relationship dynamic that can be supportive in some respects and limiting in others. The integration therapist must understand the worldview of the patients' community, the extent to which the patient subscribes to that worldview, and the impact of creating a therapeutic relationship within that context.

#### Goals and Obstacles

Cooperating with the patient to integrate meaningful psychedelic experiences involves identifying goals and obstacles for integration during assessment, and gauging how the patient defines successful integration. Even with adequate planning for integration, challenging experiences can arise that can be worrying for both the patient and therapist (Carbonaro et al., 2016; Haijen et al., 2018). However, therapists can learn to recognize and assist in managing these challenges.

### Patients Seeking Access to Psychedelic Compounds

The patient seeking a psychedelic experience may wish to discuss where to obtain psychedelics with a licensed therapist. However, in the US, most psychedelics remain designated as Schedule I drugs under the US Drug Enforcement Administration. In several other countries, certain naturally occurring psychedelics are unregulated and legal for non-medical consumption or ceremonial use by specific religious groups (Labate and Feeney, 2012). These legal backgrounds allow for the offering of psychedelics to non-Americans as part of psychedelic retreat programs, which may or may not be offering them as a healthcare service, with varying levels of legality and local government sanction (The International Center for Ethnobotanical Education, Research, and Service (ICEERS), 2020). A referral or recommendation by a licensed therapist implies the existence of an established healthcare treatment or service, and therapists are responsible for knowing and staying within the boundaries of their scope of practice (Jade, 2018). Further research and approval of psychedelics for medical use are necessary before proper, ethical recommendations or referrals can be made.

### Motivation, Knowledge, and Psychoeducation

In PHRI, preparation for a psychedelic experience is guided by a patient's reasons for using a psychedelic. The therapist must inquire about the nature of the patient's motivations. Whether they reflect an interest in spiritual growth, symptom reduction, enhancement of wellness, or a desire to enhance creativity, knowing the context and how the patient is going to use psychedelics will shape treatment and is vital for appropriate clinical care. Preparation does not imply that a person will use a psychedelic, but rather it helps a patient engage in a process of inquiry so they can make an appropriate decision for themselves. Preparation can address misinformation, dispel myths, and supply necessary education about psychedelics, making the patient's journey smoother and most importantly, safer.

Assessing a patient's level of knowledge about psychedelics and providing appropriate information and resources are essential elements of a therapeutic preparation for a psychedelic experience. Upon initial assessment, therapists can provide vital information about the basic effects of key drugs, e.g., subjective effects, physiological effects and risks, mechanisms of action, expected duration and intensity, and importantly, side effects and contraindications. This part of PHRI is based on a psychoeducational approach that aims to increase the patient's knowledge of and insight into the issue at hand (Xia et al., 2011). The main ways of achieving this are the exchange of information between the patient and the therapist, and the active communication of information (Bäuml et al., 2006). A well-informed patient is better prepared for the intervention, which is associated with achieving a more positive therapeutic outcome (Bäuml et al., 2006; Xia et al., 2011).

### Working Within the Scope of One's Practice During Preparation

When providing PHRI, it is paramount to work within the professional scope of one's practice. It is appropriate and necessary for a therapist trained in substance use treatment to talk to the patient about the dangers of certain drugs and drug-use-related practices in the context of reducing drug harm. The content of this conversation regarding specific medication interactions or health risks can vary depending on whether the therapist is a psychotherapist, prescribing therapist, or another kind of mental health practitioner. It is especially important that the therapist clearly delineate between harm reduction practices for non-clinical psychedelic use that the patient chooses on their own and a recommendation or referral for the use of a psychedelic as a mental health treatment.

### Person-Centered and Non-judgmental Approach

In the initial discussion, PHRI patients are given ample information regarding the potential risks and benefits associated with psychedelic use. As in motivational approaches to substance use treatment, the therapist aims to understand why the patient wants to have a psychedelic experience, how they plan to achieve it, what steps they envision in order to get there, and why their goal is important to them (Miller and Rollnick, 2013). It is important for the therapist to inquire whether the patient has considered alternative options, and to ask whether they are willing to receive information about alternative methods for accessing NOSCs such as holotropic breathwork (Grof and Grof, 2010). The therapist does not direct the patient toward any particular outcome, but instead provides a supportive and non-judgmental container for the patient's exploration (Miller and Rollnick, 2013). The clinician should neither encourage nor discourage the patient from psychedelic use unless it is clear that their intended use might result in harm, in which case concerns should be voiced without attaching judgement of the patient's choice.

### Psychedelics as a Non-specific Amplifiers and the Importance of Set and Setting

Additional helpful foci of preparation include introducing the idea of a psychedelic as a non-specific amplifier. This notion is based on the observation that psychedelics tend to intensify mental phenomena and amplify their significance, presenting them as being larger and more dramatic than they would have been without psychedelics (Grof, 1994; Hartogsohn, 2018). It is thought that this leads to a “manifestation of otherwise latent psychological processes” (Grof, 1994, p. 11), an increase in magical thinking (Carhart-Harris, 2013), and meaning-enhancement that is crucial in therapeutic work (Hartogsohn, 2018). As with any substance, the concept of *Set and Setting* plays an important role in psychedelic experiences; set here refers to intentions, expectations and the experiencer's overall state of mind, while setting refers to the environment in which psychedelic use takes place (World Health Organization, 1958; Leary et al., 2000; Hartogsohn, 2016, 2017). Some also use the term *cast* to describe the people present during the psychedelic experience: therapists, shamans, peers, or other ceremony participants. It is important to prepare the patient for the fact that aspects of their experience may be amplified, and that the quality of their experience is largely contingent on the set, setting, and cast of a psychedelic event. These factors are crucial in forming the experience, and their influence on the experience can increase as an effect of psychedelics (Hartogsohn, 2018).

Meaning can also be amplified during a psychedelic experience (Hartogsohn, 2018). This effect can be very useful in therapy as well as increase creativity (Baggott, 2015) and spirituality (Móró et al., 2011; Kuypers et al., 2016). Due to their meaning-enhancing aspect, psychedelic-induced revelations tend to feel more meaningful than non-psychedelic ones. This can lead to a decrease in self-inhibition and self-criticism, and a heightened focus on creativity. However, the meaning of the resulting creative achievements can also be amplified while under the influence of psychedelics or shortly thereafter (Hartogsohn, 2018). Discussing these aspects in advance can better prepare the patient for a NOSC and help their meaning-making during and after the experience.

### Self-Regulation Tools and Expectation Management

In preparation work, it is important to address the post-acute effects that a patient may encounter during a psychedelic experience. These may include general mental readiness for life after a psychedelic experience, identifying signs that may indicate the patient's need for more help, and a re-entry plan for ordinary states of consciousness and daily activities. A psychedelic-drawn NOSC can work as a powerful catalyst, stirring up deep mental and emotional issues (Bache, 1991). When a patient is in this state, trauma processing can be done in either planned or spontaneous ways (Feduccia and Mithoefer, 2018). Preparing a detailed re-entry plan in advance can help to integrate such experiences.

As with any trauma work, equipping the patient with appropriate self-regulation tools in advance can make all the difference in managing trauma symptoms (Cloitre et al., 2002; Ford and Russo, 2006). Such tools can include mindfulness practice, yoga, reflection, journaling, and guided meditation, as well as grounding practices and self-care to help the patient focus on the world outside rather than the inner world of negative feelings through concentrating on bodily sensations, arising feelings, and the surrounding environment (Najavits, 2002). As a result, the patient's focus shifts from trauma symptoms to self-reflective processing (Ford and Russo, 2006). Planning for time to rest and recuperate after a psychedelic experience can help facilitate processing and integration by giving the patient time to reflect on their insights. After the drug effects wear off, the therapist and patient should discuss any achieved insight and the content of the NOSC experience (Johnson et al., 2008).

#### Managing Expectations

Expectation management is particularly important in preparation, any disappointment felt by the patient can inform their perception of the entire psychedelic experience. Such disappointment can be related to a mismatch between expectations and outcomes. Among the frequently sought-after experiences is the *mystical experience*: a NOSC that includes a unitive quality (being one with the universe), a feeling of awe or sacredness, ineffability (the impossibility of expressing one's experience in words), transcendence of time and space, a noetic quality (sense of revelation), and a deep positive mood (Pahnke, 1969; MacLean et al., 2012). A main feature of a mystical experience is a unitive quality related to ego-dissolution (Hood, 1975; Stace, 1987; James, 2008; Nour et al., 2016). Experiencers have reported a psychedelic-induced mystical-type experience as being one of the most important events of their life (Griffiths et al., 2008). Such experiences have been positively correlated with a long-lasting increase in well-being (Barrett et al., 2015), openness (MacLean et al., 2011), and decreases in various kinds of symptomatology (Johnson et al., 2019), and are thus commonly sought after by psychedelics users (Pollan, 2018; Carey, 2019). However, not every instance of psychedelic use will result in a mystical-type experience (Majić et al., 2015; Elsey, 2017), and a strong desire for such an experience can lead to a deep disappointment in its absence. Working with this sense of disappointment or other undesired outcomes is a key element of PHRI.

### Adverse Psychedelic Experiences and Experiential Processing

When using psychedelics, an individual may have a difficult experience such as panic, anxiety, and psychotic-like reactions (Carhart-Harris, 2013). Positive and recreational intentions have been found to predict a lower likelihood of a difficult experience, while higher doses have been associated with a higher prevalence of both mystical-type and difficult experiences (Haijen et al., 2018). Uncontrolled settings and the personality trait of neuroticism predict challenging psychedelic experiences (Barrett et al., 2017). In naturalistic settings, the intensity of difficult experiences correlates positively with beneficial long-term effects, while their duration correlates negatively with such effects (Carbonaro et al., 2016). However, the majority of those who have a challenging psychedelic experience recognize a long-lasting positive effect on their well-being (Carbonaro et al., 2016).

When discomfort arises during a challenging psychedelic experience, it can be helpful for the individual to stay with the discomfort and remain curious to what is arising. This is best conveyed by the technique of *Experiential Processing*, which is “characterized by direct, non-judgmental and concrete awareness of sensory and bodily experience as it unfolds moment-to-moment” (Gadeikis et al., 2017, p. 69). Staying with the experience of discomfort can facilitate moving through the adverse reaction. A therapist can be instrumental in advising the patient on how to get reassurance, how to stay safe throughout the experience, and ways to traverse it.

### Diagnosis of Adverse Reactions

The DSM-5 (American Psychiatric Association, 2013) recognizes psychedelic use only as it pertains to Hallucinogen Persisting Perception Disorder, hallucinogen use disorders, and hallucinogen-induced disorders. Anecdotal observations by therapists working with patients who use psychedelics have reported the limitation of the DSM-5 diagnostic system in capturing the adverse reactions to psychedelics, such as anxiety, worsened moods, psychological trauma-like symptoms, or somatic symptoms. Clinicians should be aware of the diagnostic criterion and categories related to psychedelic use, and apply them where justified and appropriate, while also considering the psychological, developmental, and environmental factors that may have contributed to the emergence of these symptoms.

## Integration

Psychedelic integration is a process in which the patient integrates the insights of their experience into their life, and PHRI is a method of supporting that in the clinical consultation room. As such, the PHRI therapist may encounter a range of challenges and therapeutic tasks while working with patients who are moving through this phase. Psychotherapy sessions that occur after a psychedelic experience and are intended to work with that experience are often called *integration sessions*; however, the integration process is grounded and reflected in what occurs before, during, and outside of these sessions. Here, we cover several considerations for this stage of therapeutic encounter.

### Working With Challenging Experiences

Patients and clinicians may simultaneously experience apprehension and anxiety when dealing with difficult psychedelic experiences. Psychological symptoms after a psychedelic experience can take the form of anxiety, panic, and depressed mood (Johnson et al., 2008; Carbonaro et al., 2016). Distress can be accompanied by patient beliefs that these symptoms will persist for the remainder of their lives. For clinicians, anxiety may stem from interpreting psychological symptoms as early indicators of severe mental illness or the loss of orientation to reality. To reassure the patient and avoid overreaction to distress, clinicians should refrain from such premature conclusions while staying informed about psychedelics, their effects, and their risks.

#### Long-Term Mental Health Outcomes

Studies have been unable to establish a link between lifetime psychedelic use and negative long-term mental health outcomes (Hendricks et al., 2015a,b; Johansen and Krebs, 2015; Garcia-Romeu et al., 2019). In most cases, symptoms of distress following psychedelic use resolve without the development of a chronic psychiatric disorder. Symptoms of distress do not necessarily block beneficial psychological outcomes, especially if appropriate safeguards are implemented to minimize the risk of psychological harm (Johnson et al., 2008). However, after a psychedelic's pharmacological duration of action has ended, persisting symptoms such as insomnia, disorientation, anxiety, and depression indicate a patient in need of further psychotherapeutic support. Clinicians must distinguish between psychological symptoms that can be managed through psychological support and more severe reactions such as a psychotic episode resulting from pre-existing vulnerabilities (Heriot-Maitland, 2008; Johnson and Friedman, 2008).

#### Traumatic Experiences

Some patients who seek PHRI have experienced a negative or even traumatic encounter with psychedelics, when used outside of clinical trials. For example, regarding psilocybin use, certain cognitive states (e.g., preoccupation and confusion) are correlated with memorable, adverse experiences linked to feelings of discomfort, vulnerability, and fear (Russ et al., 2019). Individuals who use ayahuasca have reported reliving memories of past events, and thus may be at risk for re-traumatization if these past events were traumatic in nature (Nielson and Megler, 2014; Kaasik and Kreegipuu, 2020). Clinicians should reassure patients that others have encountered similar experiences and recovered from them. Such assurances contribute to the normalization of difficult experiences and reduce associated anxiety. When the intensity of the experience has subsided such that the patient is able to safely engage in discussion, the clinician can facilitate the exploration of the meaning of the experience.

The importance of a difficult or traumatic experience and a patient's related insights should be validated and met with compassion by the integration therapist; a patient should not be blamed or made to feel they are at fault for their difficult experience. It should also be acknowledged that as patients develop new personal meanings or understandings of themselves and their interpersonal relationships, they may experience increased psychological distress while simultaneously experiencing a decrease in experiential avoidance. By encountering previously avoided psychological content through the use of psychedelics, patients may begin to realize what preceded their avoidance. These realizations should be incorporated into the integration process.

#### Challenging Experiences With Positive Outcomes

In a survey of nearly 2,000 individuals who had an adverse experience with psilocybin outside of clinical trial contexts, over one-third reported the experience to be one of the most challenging events of their lifetime. Despite their difficult experiences, 84% reported positive, long-term outcomes (Carbonaro et al., 2016). Thus, many individuals have benefited from psychedelic experiences on their own. However, individuals in clinical trials who were administered doses of psilocybin for treatment-resistant depression without adequate external support systems reported feeling alienated and vulnerable after preparation and dosing sessions, and stated that they would have benefited from additional integration sessions (Watts et al., 2017). Therefore, PHRI may provide extra support to individuals who are struggling to make use of challenging experiences and may increase the likelihood of positive long-term outcomes, especially when individuals may not otherwise have access to a quality support system regarding their use of psychedelics.

Therefore, while the patient may appear to be in crisis, it may be a temporary presentation of increased symptom severity that is a part of the patient's therapeutic process. This period may be counterintuitive for a clinician since the temporary worsening of symptoms differs from the traditional psychiatric model of symptom reduction. Psychedelic integration providers can provide a safe container to support their patients through challenging periods.

### Addressing Fear

Fearful reactions may be understood within the processes of the ACT framework (Hayes et al., 2006). Although PHRI does not necessitate the adoption of the ACT model, ACT offers a well-developed framework for understanding the experience and process of fear, and its language is employed here for this purpose. In this conceptualization, the anxiety or panic that can occur during an adverse encounter with psychedelics can be equated with instances of unmitigated fear (Barrett et al., 2016). Individuals may engage in experiential avoidance by attempting to control the psychedelic experience or avoid some of the experience's psychological content, resulting in psychological distress, or harm. Acceptance, rather than experiential avoidance, is aimed at increasing psychological flexibility, or “the ability to contact the present moment more fully as a conscious human being, and to change or persist in behavior when doing so serves valued ends” (Hayes et al., 2006, p. 7). Low levels of psychological flexibility are associated with higher rates of psychopathology (Leahy et al., 2012; Masuda and Tully, 2012) and lower overall quality of life (Hayes et al., 2006). Psychedelic integration, including as it pertains to fearful reactions, may be useful in increasing psychological flexibility (Watts and Luoma, 2020). In conceptualizing psychological flexibility as a component of integration, clinicians can address fear by examining it in detail and subsequently encouraging the acceptance and awareness of feelings or thoughts that underlie fearful reactions.

Clinicians should keep in mind that there are distinguishable meanings in the fears experienced during an encounter with psychedelics. Aspects of the psychedelic experience are not purely random, and are a reflection of an individual's biopsychosocial factors. Fear, a form of psychological content, can be reflective of an individual's biographical history, wishes, and desires. Psychological content is also shaped by individual mindset (set), as well as physical and cultural environmental influences (setting). In a psychedelic experience, psychological distress can be amplified to fear or intense panic, paranoia, or loosened connection to reality. Negative or traumatic experiences may be linked to the amplification of an individual's undesired thoughts, feelings, or autobiographical memories. The amplification of psychological distress triggers experiential avoidance, and at times, a biological reductionist view in which the patient attributes their distress to the psychedelic drug alone rather than to a drug-individual interaction or the interaction between the psychedelic, their psychology, and life circumstances.

Many individuals, particularly in the absence of preparation, find it difficult to understand or communicate their psychedelic experiences, and may not realize the potential for therapy to aid in the organization and transformation of these experiences. This leaves room for integration therapists “to help bridge the gap between the lessons of the psychedelic experience and the ongoing life circumstances” (Watts and Luoma, 2020, p. 98). This entails engaging the patient to help them understand why the negative experience arose and how they can grow from it.

#### Ego Dissolution

Psychedelics reportedly trigger cognitive phenomena such as ego dissolution and ontological shock, which can incite or exacerbate fear responses (Nour et al., 2016; Carhart-Harris and Friston, 2019). Nour et al. (2016) define ego dissolution as “the experience of a compromised sense of self,” (p. 2) (2) while ontological shock entails being forced to abruptly shift one's worldview. The impact of these phenomena can be understood in terms of an interaction between the psychedelic drug and individual circumstances. Taves (2020) called for updated measures to gauge the flexible attributes of ego-dissolution, as previous research has shown the experience of ego dissolution to be influenced by set and setting, as well as drug dose. Consequently, clinicians should pay mind to the extant drug, setting, and individual interaction when encountering patients who have experienced ego dissolution or ontological shock, regardless of whether the patient reports the experience to be helpful or fearsome, because these phenomena can occur abruptly, be disorienting, and result in a need for therapeutic support.

### Increase in Sensitivity

If negative response to stress is conceptualized within the ACT framework, individuals can be seen as adopting psychologically inflexible processes to avoid discomfort within certain contexts (Hayes et al., 2006). This is somewhat similar to the idea within psychodynamic theory of adoption of ego defenses or defense mechanisms as a function of individual adaptation to one's environment (Paulhus et al., 1997). In each case, psychopathology results, in part, from the overuse or rigid application of certain processes and a lack of psychological flexibility. Psychedelics may help individuals become aware of the ways they have historically coped with stressors in their environment, and how they have suffered from psychological inflexibility which has interfered with their desire to live life openly and true to their values.

Psychedelics are hypothesized to increase psychological flexibility, leaving individuals to feel more open and less avoidant by activating mindfulness, acceptance, commitment, and behavior change processes central to ACT theory (Davis et al., 2020). Emotions become more available, when previously they may have been numbed or protected by psychological defensiveness. However, as defensiveness is reduced, these newfound feelings of vulnerability and sensitivity can lead to increased fear and anxiety, requiring therapeutic assistance to navigate. To construct meaning out of the psychedelic experience is to assist in the advancement through the period of increased vulnerability which occurs during the unfolding process.

### The Unfolding Process

The *unfolding process*, a concept borrowed from humanistic psychology, signals the continuous unraveling of insights about oneself and one's relationships after a psychedelic experience, which can take place over the span of weeks or months. Welwood, (1982 p. 92) described two types of unfolding processes. In sequential or *horizontal unfolding*, new personal meanings develop progressively over time, with each meaning building on previous meanings. In *vertical unfolding*, new, radical personal meanings emerge extemporaneously, along with the subjective experience of an increased depth of reality. Due to the continuous pursuit of the construction of meaning and the propensity for unanticipated outcomes following a psychedelic experience, both unfolding processes are essential in understanding the subjective experience of the aftermath of psychedelic use.

During unfolding, the manifestation and intensity of symptoms may fluctuate in waves, while simultaneously presenting as one of the most difficult stages of the therapeutic process. After the acute pharmacological effects of the psychedelic have worn off, patients may experience increased vulnerability, sadness, and anger. Individuals may also experience a *body/mind knowing* in which they experience feelings or vague meanings about difficult psychological events that they cannot fully articulate or realize (Welwood, 1982). Therefore, it is not surprising that during these unfolding processes, individuals may turn to an integration therapist for help. The integration therapist supports the patient to work through the unfolding process by helping the patient focus inward or ground themselves, making implicitly felt meanings explicit, addressing physiological stress, and encouraging the development of psychologically flexible coping skills.

The unfolding process coincides with the concept of psychological flexibility in the ACT model, and thus can be construed from a contextual behavioral perspective. The therapists role is to ensure the expansion of patients' awareness by reducing experiential avoidance or attempts to change unwanted affective responses (Levenson, 1976; Welwood, 1982). Therapeutic unfolding involves sitting with and accepting experiences and affective responses, allowing experiences to arise and pass, and extending beyond intellectual analysis in the pursuit of highlighting valued meanings. During the process, it may be helpful to pay particular attention to the body and the utilization of somatic therapies.

### Other Challenges During Integration

Mismatch between expectation and the actual psychedelic experience often results in a focus on comparing and contrasting what was expected and what actually occurred, which distracts attention from important, novel aspects of the actual experience. Expectations can include the belief that one must have a mystical experience to benefit from psychedelics, or that having a psychedelic experience is a panacea for ongoing psychological issues. When patients do not obtain the outcome they seek, feelings of hopelessness may arise, along with the idea that if psychedelics cannot help them, nothing can. Patients may also attribute the absence of a mystical experience to spiritual deficiency. Disappointment can be understood as a manifestation of this phenomena, and is best addressed preemptively in preparation by cultivating openness to all/actual experiences and setting aside expectations for specific experiences.

The emotional impact of disappointment may be extreme, and therapists' relational support, understanding, and compassion for patients in this experience is critical. A framework for understanding its origins and assisting patients in noticing what insights may be available to them in light of their actual experience, even if disappointing, can help guide therapeutic work toward a meaning-making process in which such experiences are opportunities for growth. The therapist should create room for the patient to discuss their disappointment without becoming defensive about the efficacy of psychedelics.

In addition to creating a container for patients to address their unmet expectations, psychoeducation can be effective in reframing disappointment. Therapists can rely on scientific literature to clarify the potential and limitations of psychedelic treatments. If patients are aware of the positive therapeutic outcomes associated with psychedelics that are reported in research or the media, the therapist should emphasize that the context of the patient's own psychedelic experience may depart dramatically from the therapeutic context provided in clinical trials. In such trials, it is often the case that participants are administered psychedelics on more than one occasion, and there is currently insufficient evidence to suggest that one psychedelic experience alone can produce long-term therapeutic outcomes (Krupitsky et al., 2007; Oehen et al., 2013; Carhart-Harris et al., 2017). It is likely that yet unidentified biological factors play a role in blunting the effect of psychedelics; additional medications can interact with psychedelics and decrease their effects (Quednow et al., 2012; Kometer et al., 2013; Kuypers, 2019).

#### Spiritual Bypass

Individuals disappointed with the outcome of psychedelic use may seek additional psychedelic experiences either by taking a higher dose or an alternative psychedelic. Clinicians should be aware of the possibility of *spiritual bypass*: engagement in spiritual practices to avoid pain and unresolved psychological distress. Put another way, spiritual bypassing encompasses attempts “to avoid or prematurely transcend basic human needs, feelings, and developmental tasks” through activities intended for self-healing or exploration such as using psychedelics, meditating, or seeking mystical experiences (Welwood, 1982, p. 64). As spiritual bypassing tends not to include processes such as acceptance and awareness, it presents openings for psychological inflexibility and thus interferes with therapeutic progress.

### The Role of the Body

It is helpful to recognize the experience of psychological difficulties in terms of stress felt in the body (Grabbe and Miller-Karas, 2018). As such, the embodiment and integration of a psychedelic experience involves the incorporation of somatic work. Somatic interventions increase awareness of sensory experiences and understanding of the relationship between physical states and psychological or emotional content. Somatic work is compatible with a variety of therapeutic modalities, but unlike traditional cognitive and behavior therapies, it focuses on a bottom-up approach in which attention is given to internal and external body sensations as well as automatic responses to stress (Grabbe and Miller-Karas, 2018). Somatic work enables patients to safely process their emotional, physiological, and psychological responses without judgment or overemphasis on the cognitive aspects of psychological distress.

The clinician can address somatic work through somatic psychotherapy, educating the patient on the importance of body-related activities, and encouraging the patient to engage in such activities outside of therapy. More specifically, patients can explore actions that promote bodily awareness, self-soothing, and body engagement such as yoga, grounding exercises, and mindfulness practices (Ostafin et al., 2006; Burrows, 2013; Myrick et al., 2015; Cushing and Braun, 2018). Bodily awareness may also be achieved through sustained attention to breathing during meditation (Millière et al., 2018). Body-related activities have repeatedly been shown to correlate with psychological well-being and increased body awareness (Carmody and Baer, 2008; Tihany et al., 2016; Van De Veer et al., 2016).

### Working With Positive Experiences

Positive psychedelic experiences can be as valuable as challenging ones. When a patient encounters a pleasurable psychedelic experience, the patient and therapist should refrain from reducing the experience to hedonistic fascination. Clinicians should also be mindful of their own potentially countertransference reactions, such as envy or disbelief. This can occur when clinicians overvalue their own role as an agent in a patient's psychological or therapeutic progress or are overly reliant on personal experience with psychedelics as a source of knowledge about the effects. Clinicians must be aware of their internal reactions to patients' subjective experiences with psychedelics, whether positive or negative.

#### Afterglow

Some individuals report a subjective positive experience of *afterglow* after a psychedelic experience, which may include heightened mood, psychological flexibility, openness, or mindfulness (Sampedro et al., 2017; Murphy-Beiner and Soar, 2020). This may relate to a period of increased neuroplasticity or a *neuroplastic window* after experiencing the acute effects of a psychedelic (Wilkinson et al., 2019). During this period, cognition, and behavior may be more susceptible to the influence of novel insights gained by the individual (Majić et al., 2015; Garcia-Romeu and Richards, 2018). Thus, the afterglow period may enable patients to explore new behaviors and ways of thinking, while aiding the transition into maintaining long-term benefits after the unfolding process.

#### Sudden Wellness

Following a psychedelic experience, patients may be highly motivated to make major life changes, such as feeling empowered to leave an undesirable employment position. The therapist should help the patient evaluate the consequences of decisions that may be ultimately helpful to the patient, but impulsive in the short-term. Moreover, rapid behavioral changes or *sudden wellness* can confuse or unsettle individuals in the patient's family and social network, especially if some among them are not ready to accept the patient's new way of being. When this occurs, it may be helpful to apply a family systems perspective, in which there is a tendency for a family to maintain equilibrium and homeostasis that is threatened when one family member experiences rapid improvement and change (Hargrove, 2009). This can contribute to particularly dramatic disequilibria in the family system. The PHRI therapist can assist patients in navigating family dynamics such that a new homeostasis is reached; one supportive of the patient's positive changes.

### Tools for Maintaining Benefits

The process of constructing meaning out of psychedelic experiences is not isolated to the therapy setting. It is the therapist's responsibility to encourage the patient to pursue activities which help sustain focus on the psychedelic experience and any resulting insights. Such activities could include journaling, meditation, artistic expression, and any other activity that aids in moving the awareness gained through psychedelic experience from an intellectual framework to a holistic framework that incorporates the body. This method of extending psychedelic integration ensures that experiences are not a momentary, fleeting state, but are embodied to facilitate long-term change.

Extending integration also involves incorporating insights gained from psychedelic experiences and integration therapy in accordance with one's values and goals. In some cases, patients will realize the need to address past traumas or ongoing psychological issues, and thus seek further structure and treatment in another form of specialized or intensive therapy. For individuals who struggle with interpersonal conflict and lack of support, this may require seeking new avenues for support and expanding one's community. Individuals may also benefit from changes in the scope of daily living, such as adjusting a career path, diet, or living arrangements to better suit their needs. The full range of possibilities for activities that extend integration cannot be covered in the scope of this paper. However, it should be noted that recommendations made by therapists should be symptom- or context-specific, or in other words, tailored to the patient's problems and concerns.

## Discussion

Patients are currently using psychedelics and seeking and receiving psychedelic integration services from clinicians, however, to date there is no published peer-reviewed literature defining psychedelic integration and no cohesive, guiding model for clinicians to draw on in this process. This paper represents the first of such models to be defined and differentiated in the academic literature.

### Summary

PHRI incorporates elements of psychedelic-assisted therapy, Mindfulness-based therapies, relational psychodynamic approaches, and IHRP to create a framework for working with patients who present either in the decision-making process prior to psychedelic use or afterward for assistance from a clinician. The PHRI model offers a departure from abstinence-based approaches to working with people who use psychedelics, enriches the therapeutic process by offering new perspectives on the decision making and meaning-making processes, and acknowledges and works with the variety of relationships patients have to psychedelics, from harmful to fully beneficial.

### Implications

Implications of the development and use of the PHRI model are broad and will continue to emerge with time. Here we identify two particularly salient implications related to the current context of this work. First, the very existence of the PHRI model and its practice indicates an acknowledgment that psychedelic use outside of clinical contexts has the potential for benefit. This challenges dominant abstinence-based paradigms that unilaterally discourage, prohibit, or stigmatize drug use. Second, the PHRI model places the clinician in the role of supporting and following the patient's preferences in a way that is aligned with harm reduction principles and psychedelic-assisted therapy, and represents a shift away from the traditional role of the clinician as the expert, authority, and giver of advice. This has implications for the distribution of power and challenges the potentially oppressive dynamics of traditional patient-clinician relationships.

As non-clinical psychedelic use increases and psychedelic-assisted therapy gains mainstream acceptance, it is critical that therapists have a model for working with patients who are considering using or have used psychedelics. Traditional paradigms and clinical training offer little more than the potential for diagnosing DSM-defined negative consequences or assessing and treating substance use disorders. PHRI offers an ethical, balanced, and theoretically grounded manner of integrating conversations about psychedelics into the clinician's existing practice while respecting the patient's unique needs and varied motivations, and as such is an important contribution to the field.

### Limitations

The objective of this paper is to set a theoretical foundation for PHRI. Due to the lack of existing literature on this topic, there is a dearth of empirical evidence for PHRI. One of the broadest limitations of PHRI is the lack of consensus regarding standards of practice and training for therapists who engage in it. Future research should focus on establishing and refining standards, and evaluating the efficacy of this approach when working with people who are contemplating or using psychedelics. The benefits of PHRI may be better tailored and administered to a larger variety of individuals if research is increased to delineate mechanisms of change, potential therapeutic outcomes, and cultural or contextual factors which influence preparation and integration.

## Conclusions

This paper has presented a transtheoretical model of PHRI which can be integrated with a variety of psychotherapeutic approaches and treatment goals. PHRI has the potential to reduce psychedelic-related harm through education and informed decision-making, as well as enhance benefit from psychedelic experiences by capitalizing on the various hypothesized mechanisms of action including increased psychological flexibility and the neuroplastic window of opportunity for change that psychedelics enable. Working in this manner, clinicians have the opportunity to incorporate PHRI into their existing practices and serve patients who use, have ever used, or are considering using psychedelics.

## Data Availability Statement

The original contributions presented in the study are included in the article/supplementary material, further inquiries can be directed to the corresponding author/s.

## Author Contributions

IG and EN conceptualized and created the theoretical model for Psychedelic Harm Reduction and Integration, proposed the manuscript structure and content areas, wrote portions of the manuscript, and oversaw writing and content contributions from AM, KC, and JS. AM, KC, and JS conducted the literature review and wrote portions of the manuscript. All authors contributed to the article and approved the submitted version.

## Conflict of Interest

IG and EN are co-founders of Fluence. IG has received consulting compensation from MAPS Public Benefit Corporation, is an advisor to Journey Clinical, Inc. and Horizons Media, Inc. EN has received consulting compensation from MAPS Public Benefit Corporation, COMPASS Pathways, Ltd. research funding from Heffter Research Institute and Riverstyx Foundation, and is an advisor to Sansero Life Sciences and the Psychedelic Medicine Association. JS is a Founder of Journey Clinical, Inc. The remaining authors declare that the research was conducted in the absence of any commercial or financial relationships that could be construed as a potential conflict of interest.

## References

[B1] A Phase 3 Program of MDMA-Assisted Psychotherapy for the Treatment of Severe Posttraumatic Stress Disorder (PTSD). MAPS Multidisciplinary Association for Psychedelic Studies. https://maps.org/research/mdma/ptsd/phase3 (accessed March 2 2021).

[B2] Ackerman S. J. Hilsenroth M. J. (2003). A review of therapist characteristics and techniques positively impacting the therapeutic alliance. Clin. Psychol. Rev. 23, 1–33. 10.1016/S0272-7358(02)00146-0 12559992

[B3] American Psychiatric Association. (2013). Diagnostic and Statistical Manual of Mental Disorders (5th ed.) Arlington, VA. 10.1176/appi.books.9780890425596

[B4] Bache C. M. (1991). Mysticism and psychedelics: the case of the dark night. J. Relig. Health 30, 215–236. 10.1007/BF00986399 24272672

[B5] Baggott M. J. (2015). Psychedelics and creativity: a review of the quantitative literature. PeerJ PrePrints 3:e1202ve1201. 10.7287/peerj.preprints.1202v1

[B6] Barrett F. S. Bradstreet M. P. Leoutsakos J.-M. S. Johnson M. W. Griffiths R. R. (2016). The challenging experience questionnaire: characterization of challenging experiences with psilocybin mushrooms. J. Psychopharmacol. 30, 1279–1295. 10.1177/0269881116678781 27856683 PMC5549781

[B7] Barrett F. S. Johnson M. W. Griffiths R. R. (2015). Validation of the revised Mystical Experience Questionnaire in experimental sessions with psilocybin. J. Psychopharmacol. 29, 1182–1190. 10.1177/0269881115609019 26442957 PMC5203697

[B8] Barrett F. S. Johnson M. W. Griffiths R. R. (2017). Neuroticism is associated with challenging experiences with psilocybin mushrooms. Pers. Individ. Dif. 117, 155–160. 10.1016/j.paid.2017.06.004 28781400 PMC5540159

[B9] Bäuml J. Froböse T. Kraemer S. Rentrop M. Pitschel-Walz G. (2006). Psychoeducation: a basic psychotherapeutic intervention for patients with schizophrenia and their families. Schizophr. Bull. 32, S1–S9. 10.1093/schbul/sbl017 16920788 PMC2683741

[B10] Bogenschutz M. P. Forcehimes A. A. (2017). Development of a psychotherapeutic model for psilocybin-assisted treatment of alcoholism. J. Human. Psychol. 57, 389–414. 10.1177/0022167816673493

[B11] Bogenschutz M. P. Forcehimes A. A. Pommy J. A. Wilcox C. E. Barbosa P. C. Strassman R. J. (2015). Psilocybin-assisted treatment for alcohol dependence: a proof-of-concept study. J. Psychopharmacol. 29, 289–299. 10.1177/0269881114565144 25586396

[B12] Bowen S. Chawla N. Witkiewitz K. (2014). “Mindfulness-based relapse prevention for addictive behaviors,” in Mindfulness-Based Treatment Approaches (Second Edition), eds Baer R. A. (San Diego, CA: Academic Press), 141–157. 10.1016/B978-0-12-416031-6.00007-4

[B13] Burrows C. J. (2013). Acceptance and commitment therapy with survivors of adult sexual assault: a case study. Clin. Case Stud. 12, 246–259. 10.1177/1534650113479652

[B14] Carbonaro T. M. Bradstreet M. P. Barrett F. S. MacLean K. A. Jesse R. Johnson M. W.. (2016). Survey study of challenging experiences after ingesting psilocybin mushrooms: acute and enduring positive and negative consequences. J. Psychopharmacol. 30, 1268–1278. 10.1177/0269881116662634 27578767 PMC5551678

[B15] Carey B. (2019, April 04). Johns hopkins opens new center for psychedelic research. The New York Times. Available online at: https://www.nytimes.com/2019/09/04/science/psychedelic-drugs-hopkins-depression.html (accessed December 20, 2020).

[B16] Carhart-Harris R. (2013). Psychedelic drugs, magical thinking and psychosis. J. Neurol. Neurosurg. Psychiatry 84:e1. 10.1136/jnnp-2013-306103.17

[B17] Carhart-Harris R. L. Friston K. J. (2019). REBUS and the anarchic brain: toward a unified model of the brain action of psychedelics. Pharmacol. Rev. 71, 316–344. 10.1124/pr.118.017160 31221820 PMC6588209

[B18] Carhart-Harris R. L. Roseman L. Bolstridge M. Demetriou L. Pannekoek J. N. Wall M. B.. (2017). Psilocybin for treatment-resistant depression: fMRI-measured brain mechanisms. Sci. Rep. 7:13187. 10.1038/s41598-017-13282-7 29030624 PMC5640601

[B19] Carmody J. Baer R. A. (2008). Relationships between mindfulness practice and levels of mindfulness, medical and psychological symptoms and well-being in a mindfulness-based stress reduction program. J. Behav. Med. 31, 23–33. 10.1007/s10865-007-9130-7 17899351

[B20] Cavnar C. (2014). The effects of ayahuasca ritual participation on gay and lesbian identity. J. Psychoactive Drugs. 46, 252–260. 10.1080/02791072.2014.920117 25052884

[B21] Clare S. (2018). Cultivating inner growth: the inner healing intelligence in MDMA-assisted psychotherpay. MAPS Bull. 28, 30–33. Available online at: https://maps.org/news/bulletin/articles/435-maps-bulletin-winter-2018-vol-28-no-3/7515-cultivating-inner-growth-the-inner-healing-intelligence-in-mdma-assisted-psychotherapy-winter-2018

[B22] Cloitre M. Koenen K. C. Cohen L. R. Han H. (2002). Skills training in affective and interpersonal regulation followed by exposure: a phase-based treatment for PTSD related to childhood abuse. J. Consult. Clin. Psychol. 70, 1067–1074. 10.1037/0022-006X.70.5.1067 12362957

[B23] Connors G. J. Carroll K. M. DiClemente C. C. Longabaugh R. Donovan D. M. (1997). The therapeutic alliance and its relationship to alcoholism treatment participation and outcome. J. Consult. Clin. Psychol. 65, 588–598. 10.1037/0022-006X.65.4.588 9256560

[B24] Coppock T. E. Owen J. J. Zagarskas E. Schmidt M. (2010). The relationship between therapist and client hope with therapy outcomes. Psychotherap. Res. 20, 619–626. 10.1080/10503307.2010.497508 20714970

[B25] Crane R. S. Stanley S. Rooney M. Bartley T. Cooper L. Mardula J. (2015). Disciplined improvisation: characteristics of inquiry in mindfulness-based teaching. Mindfulness 6, 1104–1114. 10.1007/s12671-014-0361-8 26379795 PMC4562992

[B26] Cushing R. E. Braun K. L. (2018). Mind–body therapy for military veterans with post-traumatic stress disorder: a systematic review. J. Alternat. Complement. Med. 24, 106–114. 10.1089/acm.2017.0176 28880607

[B27] Dalgarno P. Shewan D. (2005). Reducing the risks of drug use: the case for set and setting. Addict. Res. Theory 13, 259–265. 10.1080/16066350500053562

[B28] Davis A. K. Barrett F. S. Griffiths R. R. (2020). Psychological flexibility mediates the relations between acute psychedelic effects and subjective decreases in depression and anxiety. J. Contextual Behav. Sci. 15, 39–45. 10.1016/j.jcbs.2019.11.004 32864325 PMC7451132

[B29] Dore J. Turnipseed B. Dwyer S. Turnipseed A. Andries J. Ascani G.. (2019). Ketamine assisted psychotherapy (KAP): patient demographics, clinical data and outcomes in three large practices administering ketamine with psychotherapy. J. Psychoactive Drugs 51, 189–198. 10.1080/02791072.2019.1587556 30917760

[B30] Dorsen C. Palamar J. Shedlin M. G. (2019). Ceremonial ‘Plant Medicine'use and its relationship to recreational drug use: an exploratory study. Addict. Res. Theory 27, 68–75. 10.1080/16066359.2018.1455187 31534445 PMC6749819

[B31] Elsey J. W. B. (2017). Psychedelic drug use in healthy individuals: a review of benefits, costs, and implications for drug policy. Drug Sci. Policy Law 3, 1–11. 10.1177/2050324517723232

[B32] Engler J. (1984). Therapeutic aims in psychotherapy and meditation: developmental stages in the representation of self. J. Transpersonal Psychol. 16, 25–61.

[B33] Epstein M. (1988). The deconstruction of the self: Ego and “egolessness” in Buddhist insight meditation. J. Transpersonal Psychol. 20, 61–69.

[B34] Feduccia A. A. Mithoefer M. C. (2018). MDMA-assisted psychotherapy for PTSD: are memory reconsolidation and fear extinction underlying mechanisms? Prog. Neuropsychopharmacol. Biol. Psychiatry 84, 221–228. 10.1016/j.pnpbp.2018.03.003 29524515

[B35] Feller C. P. Cottone R. R. (2003). The importance of empathy in the therapeutic alliance. J. Human. Counsel. Educ. Dev. 42, 53–61. 10.1002/j.2164-490X.2003.tb00168.x24059732

[B36] Fischman L. G. (2019). Seeing without self: discovering new meaning with psychedelic-assisted psychotherapy. Neuropsychoanalysis 21, 53–78. 10.1080/15294145.2019.1689528

[B37] Ford J. D. Russo E. (2006). Trauma-focused, present-centered, emotional self-regulation approach to integrated treatment for posttraumatic stress and addiction: Trauma Adaptive Recovery Group Education and Therapy (TARGET). Am. J. Psychother. 60, 335–355. 10.1176/appi.psychotherapy.2006.60.4.335 17340945

[B38] Frecska E. Bokor P. Winkelman M. (2016). The therapeutic potentials of ayahuasca: possible effects against various diseases of civilization. Front. Pharmacol. 7:35. 10.3389/fphar.2016.00035 26973523 PMC4773875

[B39] Gadeikis D. Bos N. Schweizer S. Murphy F. Dunn B. (2017). Engaging in an experiential processing mode increases positive emotional response during recall of pleasant autobiographical memories. Behav. Res. Ther. 92, 68–76. 10.1016/j.brat.2017.02.005 28273505 PMC5390771

[B40] Garcia-Romeu A. Davis A. K. Erowid F. Erowid E. Griffiths R. R. Johnson M. W. (2019). Cessation and reduction in alcohol consumption and misuse after psychedelic use. J. Psychopharmacol. 33, 1088–1101. 10.1177/0269881119845793 31084460

[B41] Garcia-Romeu A. Richards W. A. (2018). Current perspectives on psychedelic therapy: use of serotonergic hallucinogens in clinical interventions. Int. Rev. Psychiatry 30, 291–316. 10.1080/09540261.2018.1486289 30422079

[B42] Geller S. M. (2013). “Therapeutic presence as a foundation for relational depth,” Relational Depth: New Perspectives and Developments, eds Knox R. Murphy D. Wiggins S. Cooper M. (Basingstoke: Palgrave), 175–184. 10.1007/978-1-137-29831-7_14

[B43] Goodyear K. Haass-Koffler C. L. Chavanne D. (2018). Opioid use and stigma: the role of gender, language and precipitating events. Drug Alcohol Depend. 185, 339–346. 10.1016/j.drugalcdep.2017.12.037 29499554 PMC6097242

[B44] Grabbe L. Miller-Karas E. (2018). The trauma resiliency model: a “bottom-up” intervention for trauma psychotherapy. J. Am. Psychiatr. Nurses Assoc. 24, 76–84. 10.1177/1078390317745133 29199520

[B45] Griffiths R. Richards W. Johnson M. McCann U. Jesse R. (2008). Mystical-type experiences occasioned by psilocybin mediate the attribution of personal meaning and spiritual significance 14 months later. J. Psychopharmacol. 22, 621–632. 10.1177/0269881108094300 18593735 PMC3050654

[B46] Griffiths R. R. Richards W. A. McCann U. Jesse R. (2006). Psilocybin can occasion mystical-type experiences having substantial and sustained personal meaning and spiritual significance. Psychopharmacology 187, 268–283; discussion 284-292. 10.1007/s00213-006-0457-5 16826400

[B47] Grinspoon L. Bakalar J. B. (1981). The psychedelic drug therapies. Curr. Psychiatr. Ther. 20, 275–283. 7326971

[B48] Grof S. (1994). LSD Psychotherapy (2nd ed). Alameda, CA: Hunter House.

[B49] Grof S. Grof C. (2010). Holotropic Breathwork. Albany, NY: State University of New York.

[B50] Haijen E. C. H. M. Kaelen M. Roseman L. Timmermann C. Kettner H. Russ S.. (2018). Predicting responses to psychedelics: a prospective study. Front. Pharmacol. 9:897. 10.3389/fphar.2018.00897 30450045 PMC6225734

[B51] Hargrove D. S. (2009). “Psychotherapy based on Bowen family systems theory,” in The Wiley-Blackwell Handbook of Family Psychology, eds Bray J. H. Stanton M. (Wiley Blackwell), 286–299. 10.1002/9781444310238.ch19

[B52] Hartogsohn I. (2016). Set and setting, psychedelics and the placebo response: an extra-pharmacological perspective on psychopharmacology. J. Psychopharmacol. 30, 1259–1267. 10.1177/0269881116677852 27852960

[B53] Hartogsohn I. (2017). Constructing drug effects: a history of set and setting. Drug Sci. Policy Law 3:205032451668332. 10.1177/2050324516683325

[B54] Hartogsohn I. (2018). The meaning-enhancing properties of psychedelics and their mediator role in psychedelic therapy, spirituality, and creativity. Front. Neurosci. 12:129. 10.3389/fnins.2018.00129 29559884 PMC5845636

[B55] Hayes S. C. Luoma J. B. Bond F. W. Masuda A. Lillis J. (2006). Acceptance and commitment therapy: model, processes and outcomes. Behav. Res. Ther. 44, 1–25. 10.1016/j.brat.2005.06.006 16300724

[B56] Henderson N. L. Dressler W. W. (2017). Medical disease or moral defect? Stigma attribution and cultural models of addiction causality in a university population. Cult. Med. Psychiatry 41, 480–498. 10.1007/s11013-017-9531-1 28378037

[B57] Hendricks P. S. Johnson M. W. Griffiths R. R. (2015a). Psilocybin, psychological distress, and suicidality. J. Psychopharmacol. 29, 1041–1043. 10.1177/0269881115598338 26395582 PMC4721603

[B58] Hendricks P. S. Thorne C. B. Clark C. B. Coombs D. W. Johnson M. W. (2015b). Classic psychedelic use is associated with reduced psychological distress and suicidality in the United States adult population. J. Psychopharmacol. 29, 280–288. 10.1177/0269881114565653 25586402

[B59] Herbeck D. M. Hser Y. I. Teruya C. (2008). Empirically supported substance abuse treatment approaches: a survey of treatment providers' perspectives and practices [Multicenter Study Research Support, N.I.H., Extramural]. Addict. Behav. 33, 699–712. 10.1016/j.addbeh.2007.12.003 18207334 PMC2373985

[B60] Heriot-Maitland C. P. (2008). Mysticism and madness: different aspects of the same human experience? Mental Health Relig. Cult. 11, 301–325. 10.1080/13674670701287680

[B61] Hood R. W. (1975). The construction and preliminary validation of a measure of reported mystical experience [Article]. J. Sci. Study Relig. 14:29. 10.2307/1384454

[B62] Inserra A. (2019). Current status of psychedelic therapy in Australia and New Zealand: are we falling behind? Austr. N. Zealand J. Psychiatry 53, 190–192. 10.1177/0004867418824018 30681004 PMC6399726

[B63] Jade R. (2018). Integrating Underground Psychedelic Use: A Cautionary Note for Licensed Health Care Providers. SSRN.

[B64] James W. (2008). The Varieties of Religious Experience: A Study in Human Nature. New York, NY: Routledge. 10.1017/CBO9781139149822

[B65] Johansen P.-Ø. Krebs T. S. (2015). Psychedelics not linked to mental health problems or suicidal behavior: a population study. J. Psychopharmacol. 29, 270–279. 10.1177/0269881114568039 25744618

[B66] Johnson C. V. Friedman H. L. (2008). Enlightened or delusional? Differentiating religious, spiritual, and transpersonal experiences from psychopathology. J. Human. Psychol. 48, 505–527. 10.1177/0022167808314174

[B67] Johnson M. Richards W. Griffiths R. (2008). Human hallucinogen research: guidelines for safety. J. Psychopharmacol. 22, 603–620. 10.1177/0269881108093587 18593734 PMC3056407

[B68] Johnson M. W. Garcia-Romeu A. Cosimano M. P. Griffiths R. R. (2014). Pilot study of the 5-HT2AR agonist psilocybin in the treatment of tobacco addiction. J. Psychopharmacol. 28, 983–992. 10.1177/0269881114548296 25213996 PMC4286320

[B69] Johnson M. W. Hendricks P. S. Barrett F. S. Griffiths R. R. (2019). Classic psychedelics: an integrative review of epidemiology, therapeutics, mystical experience, and brain network function. Pharmacol. Ther. 197, 83–102. 10.1016/j.pharmthera.2018.11.010 30521880

[B70] Kaasik H. Kreegipuu K. (2020). Ayahuasca users in Estonia: ceremonial practices, subjective long-term effects, mental health, and quality of life. J. Psychoactive Drugs 52, 255–263. 10.1080/02791072.2020.1748773 32299306

[B71] Kabat-Zinn J. (2006). Mindfulness-based interventions in context: past, present, and future. Clin. Psychol. 10, 144–156. 10.1093/clipsy.bpg016

[B72] Knudsen H. K. Ducharme L. J. Roman P. M. Link T. (2005). Buprenorphine diffusion: the attitudes of substance abuse treatment counselors. J. Subst. Abuse Treat. 29, 95–106. 10.1016/j.jsat.2005.05.002 16135338

[B73] Kometer M. Schmidt A. Jancke L. Vollenweider F. X. (2013). Activation of Serotonin 2A receptors underlies the psilocybin-induced effects on oscillations, N170 visual-evoked potentials, and visual hallucinations. J. Neurosci. 33, 10544–10551. 10.1523/JNEUROSCI.3007-12.2013 23785166 PMC6618596

[B74] Krebs T. S. Johansen P.-Ø. (2013). Over 30 million psychedelic users in the United States. F1000Research 2:98. 10.12688/f1000research.2-98.v1 24627778 PMC3917651

[B75] Krupitsky E. M. Burakov A. M. Dunaevsky I. V. Romanova T. N. Slavina T. Y. Grinenko A. Y. (2007). Single versus repeated sessions of ketamine-assisted psychotherapy for people with heroin dependence. J. Psychoactive Drugs 39, 13–19. 10.1080/02791072.2007.10399860 17523581

[B76] Kuypers K. P. C. (2019). Psychedelic medicine: the biology underlying the persisting psychedelic effects. Med. Hypotheses 125, 21–24. 10.1016/j.mehy.2019.02.029 30902145

[B77] Kuypers K. P. C. Riba J. de la Fuente Revenga M. Barker S. Theunissen E. L. Ramaekers J. G. (2016). Ayahuasca enhances creative divergent thinking while decreasing conventional convergent thinking. Psychopharmacology 233, 3395–3403. 10.1007/s00213-016-4377-8 27435062 PMC4989012

[B78] Labate B. C. Feeney K. (2012). Ayahuasca and the process of regulation in Brazil and internationally: implications and challenges. Int. J. Drug Policy 23, 154–161. 10.1016/j.drugpo.2011.06.006 21856141

[B79] Larimer M. E. Palmer R. S. Marlatt G. A. (1999). Relapse prevention. an overview of marlatt's cognitive-behavioral model. Alcohol Res. Health 23, 151–160. 10890810 PMC6760427

[B80] Lawn W. Hallak J. E. Crippa J. A. Dos Santos R. Porffy L. Barratt M. J.. (2017). Well-being, problematic alcohol consumption and acute subjective drug effects in past-year ayahuasca users: a large, international, self-selecting online survey. Sci. Rep. 7:15201. 10.1038/s41598-017-14700-6 29123145 PMC5680239

[B81] Leahy R. L. Tirch D. D. Melwani P. S. (2012). Processes underlying depression: risk aversion, emotional schemas, and psychological flexibility. Int. J. Cogn. Ther. 5, 362–379. 10.1521/ijct.2012.5.4.362

[B82] Leary T. Metzner R. Alpert R. (2000). The Psychedelic Experience: A Manual Based on the Tibetan Book of the Dead. New York, NY: Citadel Press, Kensington Publishing Corp.

[B83] Levenson E. A. (1976). A holographic model of psychoanalytic change. Contemp. Psychoanal. 12, 1–20. 10.1080/00107530.1976.10745411

[B84] MacLean K. A. Johnson M. W. Griffiths R. R. (2011). Mystical experiences occasioned by the hallucinogen psilocybin lead to increases in the personality domain of openness. J. Psychopharmacol. 25, 1453–1461. 10.1177/0269881111420188 21956378 PMC3537171

[B85] MacLean K. A. Leoutsakos J. M. Johnson M. W. Griffiths R. R. (2012). Factor analysis of the mystical experience questionnaire: a study of experiences occasioned by the hallucinogen psilocybin. J. Sci. Study Relig. 51, 721–737. 10.1111/j.1468-5906.2012.01685.x 23316089 PMC3539773

[B86] Majić T. Schmidt T. T. Gallinat J. (2015). Peak experiences and the afterglow phenomenon: When and how do therapeutic effects of hallucinogens depend on psychedelic experiences? J. Psychopharmacol. 29, 241–253. 10.1177/0269881114568040 25670401

[B87] Mangrum L. F. Spence R. T. Lopez M. (2006). Integrated versus parallel treatment of co-occurring psychiatric and substance use disorders. J. Subst. Abuse Treat. 30, 79–84. 10.1016/j.jsat.2005.10.004 16377455

[B88] Marlatt G. A. (1996). Harm reduction: come as you are. Addict. Behav. 21, 779–788. 10.1016/0306-4603(96)00042-1 8904943

[B89] Marlatt G. A. Larimer M. E. Witkiewitz K. (2011). Harm Reduction: Pragmatic Strategies for Managing High-Risk Behaviors. New York, NY: Guilford Press.

[B90] Masuda A. Tully E. C. (2012). The role of mindfulness and psychological flexibility in somatization, depression, anxiety, and general psychological distress in a nonclinical college sample. J. Evid. Based Complement. Altern. Med. 17, 66–71. 10.1177/2156587211423400

[B91] McCown D. Reibel D. Micozzi M. S. (2010). Teaching Mindfulness: A Practical Guide for Clinicians and Educators. New York, NY: Springer. 10.1007/978-0-387-09484-7

[B92] Miller W. R. Rollnick S. (2013). Motivational Interviewing: Helping People Change (3rd ed). New York, NY: Guilford Press.

[B93] Millière R. Carhart-Harris R. L. Roseman L. Trautwein F.-M. Berkovich-Ohana A. (2018). Psychedelics, meditation, and self-consciousness. Front. Psychol. 9:1475. 10.3389/fpsyg.2018.01475 30245648 PMC6137697

[B94] Mitchell S. A. (2009). Relational Concepts in Psychoanalysis. Cambridge, MA: Harvard University Press. 10.2307/j.ctvk12rmv

[B95] Mithoefer M. (2013). MDMA-Assisted Psychotherapy: How Different is it From Other Psychotherapy. MAPS Bulletin Special Edition, Berkeley, CA: Springer, 10–14.

[B96] Mithoefer M. C. Designee S. Doblin R. Emerson A. (2008). A Manual for MDMA-Assisted Psychotherapy in the Treatment of Posttraumatic Stress Disorder.

[B97] Móró L. Simon K. Bárd I. Rácz J. (2011). Voice of the psychonauts: coping, life purpose, and spirituality in psychedelic drug users. J. Psychoactive Drugs 43, 188–198. 10.1080/02791072.2011.605661 22111402

[B98] Murphy-Beiner A. Soar K. (2020). Ayahuasca's ‘afterglow': improved mindfulness and cognitive flexibility in ayahuasca drinkers. Psychopharmacology 237, 1161–1169. 10.1007/s00213-019-05445-3 31927605

[B99] Myrick A. C. Chasson G. S. Lanius R. A. Leventhal B. Brand B. L. (2015). Treatment of complex dissociative disorders: a comparison of interventions reported by community therapists versus those recommended by experts. J. Trauma Dissoc. 16, 51–67. 10.1080/15299732.2014.949020 25365637

[B100] Najavits L. (2002). Seeking Safety: A Treatment Manual for PTSD and Substance Abuse. New York, NY: Guilford Press.

[B101] Nichols D. E. (2016). Psychedelics. Pharmacol. Rev. 68, 264–355. 10.1124/pr.115.011478 26841800 PMC4813425

[B102] Nielson J. L. Megler J. D. (2014). “Ayahuasca as a candidate therapy for PTSD,” in The Therapeutic Use of Ayahuasca (Springer), 41–58. 10.1007/978-3-642-40426-9_3

[B103] Noakes J. (2019, April 14). Psychedelic Renaissance: Could MDMA Help With PTSD, Depression and Anxiety? The Guardian. Available online at: https://www.theguardian.com/science/2019/apr/14/psychedelic-renaissance-could-mdma-help-with-ptsd-depression-and-anxiety (accessed December 20, 2020).

[B104] Nour M. M. Evans L. Nutt D. Carhart-Harris R. L. (2016). Ego-dissolution and psychedelics: validation of the Ego-Dissolution Inventory (EDI). Front. Hum. Neurosci. 10:269. 10.3389/fnhum.2016.00269 27378878 PMC4906025

[B105] Nutt D. J. King L. A. Phillips L. D. (2010). Drug harms in the UK: a multicriteria decision analysis. Lancet 376, 1558–1565. 10.1016/S0140-6736(10)61462-6 21036393

[B106] Oehen P. Traber R. Widmer V. Schnyder U. (2013). A randomized, controlled pilot study of MDMA (±3, 4-Methylenedioxymethamphetamine)-assisted psychotherapy for treatment of resistant, chronic Post-Traumatic Stress Disorder (PTSD). J. Psychopharmacol. 27, 40–52. 10.1177/0269881112464827 23118021

[B107] O'Malley P. (1999). “Consuming risks: harm minimization and the government of “drug users,” in Governable Places: Readings on Governmentality and Crime Control (Ashgate), 191–214. 10.4324/9780429427114-8

[B108] Ostafin B. D. Chawla N. Bowen S. Dillworth T. M. Witkiewitz K. Marlatt G. A. (2006). Intensive mindfulness training and the reduction of psychological distress: a preliminary study. Cogn. Behav. Pract. 13, 191–197. 10.1016/j.cbpra.2005.12.001

[B109] Pahnke W. (1969). Psychedelic drugs and mystical experience. Int. Psychiatry Clin. 5:149. 4892137

[B110] Palamar J. J. Griffin-Tomas M. Ompad D. C. (2015). Illicit drug use among rave attendees in a nationally representative sample of US high school seniors. Drug Alcohol Depend. 152, 24–31. 10.1016/j.drugalcdep.2015.05.002 26005041 PMC4458153

[B111] Paulhus D. L. Fridhandler B. Hayes S. (1997). “Psychological defense: contemporary theory and research,” in Handbook of Personality Psychology (Cambridge, MA: Elsevier), 543–579. 10.1016/B978-012134645-4/50023-8

[B112] Phelps J. (2017). Developing guidelines and competencies for the training of psychedelic therapists. J. Human. Psychol. 57, 450–487. 10.1177/0022167817711304

[B113] Phelps J. (2019). Training Psychedelic Therapists. Advances in Psychedelic Medicine: State-of-the-Art Therapeutic Applications. Santa Barbara, CA: Praeger, 274.

[B114] Pickard H. (2017). Responsibility without Blame for Addiction. Neuroethics 10, 169–180. 10.1007/s12152-016-9295-2 28725286 PMC5486507

[B115] Pisano V. D. Putnam N. P. Kramer H. M. Franciotti K. J. Halpern J. H. Holden S. C. (2017). The association of psychedelic use and opioid use disorders among illicit users in the United States. J. Psychopharmacol. 31, 606–613. 10.1177/0269881117691453 28196428

[B116] Polak F. (2000). Thinking about drug law reform: some political dynamics of medicalization. Fordham Urban Law J. 28, 351–361.

[B117] Pollan M. (2018). How to Change Your Mind: What the New Science of Psychedelics Teaches us About Consciousness, Dying, Addiction, Depression, and Transcendence. New York, NY: Penguin Press. 10.1080/02791072.2018.1535149 30376643

[B118] Prayag G. Mura P. Hall M. Fontaine J. (2015). Drug or spirituality seekers? Consuming ayahuasca. Ann. Tourism Res. 52, 175–177. 10.1016/j.annals.2015.03.008

[B119] Quednow B. B. Kometer M. Geyer M. A. Vollenweider F. X. (2012). Psilocybin-induced deficits in automatic and controlled inhibition are attenuated by ketanserin in healthy human volunteers. Neuropsychopharmacology 37, 630–640. 10.1038/npp.2011.228 21956447 PMC3260978

[B120] Rieckmann T. i,. R Kovas A. E. McFarland B. H. Abraham A. J. (2011). A multi-level analysis of counselor attitudes toward the use of buprenorphine in substance abuse treatment. J. Subst. Abuse Treat. 41, 374–385. 10.1016/j.jsat.2011.05.005 21821379 PMC3486698

[B121] Ritter A. Cameron J. (2006). A review of the efficacy and effectiveness of harm reduction strategies for alcohol, tobacco and illicit drugs. Drug Alcohol Rev. 25, 611–624. 10.1080/09595230600944529 17132577

[B122] Russ S. L. Carhart-Harris R. L. Maruyama G. Elliott M. S. (2019). Replication and extension of a model predicting response to psilocybin. Psychopharmacology 236, 3221–3230. 10.1007/s00213-019-05279-z 31203401

[B123] Sampedro F. de la Fuente Revenga M. Valle M. Roberto N. Domínguez-Clav,é E. Elices M.. (2017). Assessing the psychedelic “After-Glow” in ayahuasca users: post-acute neurometabolic and functional connectivity changes are associated with enhanced mindfulness capacities. Int. J. Neuropsychopharmacol. 20, 698–711. 10.1093/ijnp/pyx036 28525587 PMC5581489

[B124] Segal Z. V. Williams J. M. G. Teasdale J. D. (2012). Mindfulness-Based Cognitive Therapy for Depression: A New Approach to Preventing Relapse (2nd ed.). New York, NY: The Guilford Press.

[B125] Sessa B. Higbed L. Nutt D. (2019). A review of 3,4-methylenedioxymethamphetamine (MDMA)-Assisted Psychotherapy. Front. Psychiatry 10:138. 10.3389/fpsyt.2019.00138 30949077 PMC6435835

[B126] Sloshower J. (2018). “Integrating psychedelic medicines and psychiatry: theory and methods of a model clinic, in Plant Medicines, Healing and Psychedelic Science, eds Labate B. C. Cavnar C. (Springer International Publishing), 113–132. 10.1007/978-3-319-76720-8_7

[B127] Snyder W. U. (1945). An investigation of the nature of non-directive psychotherapy. J. Gen. Psychol. 33, 193–223. 10.1080/00221309.1945.10544506 21006712

[B128] Stace W. T. (1987). Mysticism and Philosophy, eds Stace W. T. Tarcher J. P.. New York, NY: Distributed by St. Martin's Press.

[B129] Szott K. (2015). Contingencies of the will: Uses of harm reduction and the disease model of addiction among health care practitioners. Health 19, 507–522. 10.1177/1363459314556904 25394654 PMC4430440

[B130] Tatarsky A. (2003). Harm reduction psychotherapy: extending the reach of traditional substance use treatment. J. Subst. Abuse Treat. 25, 249–256. 10.1016/S0740-5472(03)00085-0 14693253

[B131] Tatarsky A. (2007). Harm Reduction Psychotherapy: A New Treatment for Drug and Alcohol Problems. Lanham, MD: Jason Aronson.

[B132] Tatarsky A. Kellogg S. (2010). Integrative harm reduction psychotherapy: a case of substance use, multiple trauma, and suicidality. J. Clin. Psychol. 66, 123–135. 10.1002/jclp.20666 20088005

[B133] Tatarsky A. Marlatt G. A. (2010). State of the art in harm reduction psychotherapy: an emerging treatment for substance misuse. J. Clin. Psychol. 66, 117–122. 10.1002/jclp.20672 20049922

[B134] Taves A. (2020). Mystical and other alterations in sense of self: an expanded framework for studying nonordinary experiences. Perspect. Psychol. Sci. 15, 669–690. 10.1177/1745691619895047 32053465

[B135] Taylor K. (2014). “Ethical caring in psychedelic work,” in Manifesting Minds: A Review of Psychedelics in Science, Medicine, Sex, and Spirituality, Vol. 136, eds Doblin R. Burge B. (Berkeley: North Atlantic Books).

[B136] The International Center for Ethnobotanical Education Research Service (ICEERS). (2020). Myths & Realities of Ayahuasca Legality (I). Retrieved from: https://www.iceers.org/myths-realities-of-ayahuasca-legality/ (accessed March 2, 2021).

[B137] Tihany B. T. Böor P. Emanuelsen L. Köteles F. (2016). Mediators between Yoga practice and psychological well-being: mindfulness, body awareness, and satisfaction with body image. Europ. J. Mental Health 11, 112–127. 10.5708/EJMH.11.2016.1-2.7

[B138] Treleaven D. A. (2018). Trauma-Sensitive Mindfulness: Practices for Safe and Transformative Healing. New york, NY: WW Norton & Company.

[B139] Valentino T. (2020). MDMA-Assisted Psychotherapy for PTSD: To Phase 3 and Beyond. Psych Congress 2020. Available online at: https://www.psychcongress.com/article/mdma-assisted-psychotherapy-ptsd-trials-progressing-through-phase-3 (accessed December 20, 2020).

[B140] Van De Veer E. Van Herpen E. Van Trijp H. C. M. (2016). Body and mind: mindfulness helps consumers to compensate for prior food intake by enhancing the responsiveness to physiological cues. J. Consumer Res. 42, 783–803. 10.1093/jcr/ucv058

[B141] Vollenweider F. X. Kometer M. (2010). The neurobiology of psychedelic drugs: implications for the treatment of mood disorders. Nat. Rev. Neurosci. 11:10. 10.1038/nrn2884 20717121

[B142] Watts R. Day C. Krzanowski J. Nutt D. Carhart-Harris R. (2017). patients' accounts of increased “Connectedness” and “Acceptance” after psilocybin for treatment-resistant depression. J. Human. Psychol. 57, 520–564. 10.1177/0022167817709585

[B143] Watts R. Luoma J. B. (2020). The use of the psychological flexibility model to support psychedelic assisted therapy. J. Contextual Behav. Sci. 15, 92–102. 10.1016/j.jcbs.2019.12.004

[B144] Welwood J. (1982). Principles of inner work: psychological and spiritual. J. Transpers. Psychol. 16:1984.

[B145] Wilkinson S. T. Holtzheimer P. E. Gao S. Kirwin D. S. Price R. B. (2019). Leveraging neuroplasticity to enhance adaptive learning: the potential for synergistic somatic-behavioral treatment combinations to improve clinical outcomes in depression. Biol. Psychiatry 85, 454–465. 10.1016/j.biopsych.2018.09.004 30528745 PMC6380941

[B146] Winkelman M. (2005). Drug tourism or spiritual healing? Ayahuasca seekers in amazonia [Article]. J. Psychoactive Drugs 37, 209–218. 10.1080/02791072.2005.10399803 16149335

[B147] Woods S. L. Rockman P. Collins E. (2016). A Contemplative Dialogue: The Inquiry Process in Mindfulness-Based Interventions. Centre for Mindfulness Studies, Oakland, CA. Available online at: https://mbpti.org/wp-content/uploads/2016/04/Woods-S.-L.-Rockman-P.-Collins-E.-2016-A-Complentative-Dialogue.pdf

[B148] Woods S. L. Rockman P. Collins E. (2019). Mindfulness-Based Cognitive Therapy: Embodied Presence and Inquiry in Practice. New Harbinger Publications.

[B149] World Health Organization (1958). Ataractic and Hallucinogenic Drugs in Psychiatry: Report of a Study Group [meeting held in Geneva from 4 to 9 November 1957]. Geneva: World Health Organization. 13636238

[B150] Xia J. Merinder L. B. Belgamwar M. R. (2011). Psychoeducation for schizophrenia. Schizophr. Bull. 37, 21–22. 10.1093/schbul/sbq138 21147896 PMC3004189

[B151] Yaden D. B. Le Nguyen K. D. Kern M. L. Wintering N. A. Eichstaedt J. C. Schwartz H. A.. (2017). The noetic quality: a multimethod exploratory study. Psychol. Consciousness 4:54. 10.1037/cns0000098

[B152] Yockey R. A. Vidourek R. A. King K. A. (2020). Trends in LSD use among US adults: 2015–2018. Drug Alcohol Depend. 212:108071. 10.1016/j.drugalcdep.2020.108071 32450479

